# Transport in serial spinful multiple-dot systems: The role of electron-electron interactions and coherences

**DOI:** 10.1038/srep22761

**Published:** 2016-03-07

**Authors:** Bahareh Goldozian, Fikeraddis A. Damtie, Gediminas Kiršanskas, Andreas Wacker

**Affiliations:** 1 Mathematical Physics and NanoLund, Lund University, Box 118, S-22100 Lund, Sweden

## Abstract

Quantum dots are nanoscopic systems, where carriers are confined in all three spatial directions. Such nanoscopic systems are suitable for fundamental studies of quantum mechanics and are candidates for applications such as quantum information processing. It was also proposed that linear arrangements of quantum dots could be used as quantum cascade laser. In this work we study the impact of electron-electron interactions on transport in a spinful serial triple quantum dot system weakly coupled to two leads. We find that due to electron-electron scattering processes the transport is enabled beyond the common single-particle transmission channels. This shows that the scenario in the serial quantum dots intrinsically deviates from layered structures such as quantum cascade lasers, where the presence of well-defined single-particle resonances between neighboring levels are crucial for device operation. Additionally, we check the validity of the Pauli master equation by comparing it with the first-order von Neumann approach. Here we demonstrate that coherences are of relevance if the energy spacing of the eigenstates is smaller than the lead transition rate multiplied by *ħ*.

Electron-electron interaction effects in quantum dots have been a topic of active research within the past decades^1^,^2^,^3^,^4^,^5^. The ability to confine a finite number of charged particles with advanced fabrication techniques in these structures opened up a possibility for testing different physical theories such as charge and conductance quantization^6^,^7^,^8^,^9^,^10^, Coulomb blockade^11^,^12^, exciton formation^13^,^14^, just to mention a few.

Here we focus on the electric transport through a serial arrangement of multiple quantum dots. Experimentally they can be realized in different ways and prominent examples are the gating of a two-dimensional electron gas^15^,^16^,^17^, cleaved edge overgrowth structures^18^, stacked self-organized quantum dots^19^, nanowires either with external gates^20^,^21^ or embedded heterostructures^22^, or the arrangement of atoms by a scanning tunneling microscope^23^. The restriction of phase space in such low-dimensional structures is reducing the scattering rates substantially and therefore such structures have been suggested for a wide range of applications ranging from quantum information processing^24^,^25^ to quantum cascade lasers^26^,^27^.

Electron transport through these structures has been widely used for level spectroscopy^28^,^29^,^30^,^31^. Generally, one assumes, that the transport through quantum dot systems is dominated by specific resonances. These occur due to the alignment of energy levels in individual dots with those in neighboring dots as well as with the chemical potentials of metallic leads. This provides specific conditions for transport, which are resolved as current or conductance peaks for varying external parameters, such as the voltages at different gates. Such resonances may even refer to states with different energies due to the emission of phonons with a specified frequency^32^,^33^ or Auger processes^34^. But even in this case the existence of specific resonances is the guiding theme of studying multiple dot systems. However, with increasing number of dots the number of resonance conditions becomes large and difficult to satisfy simultaneously. Taking into account growth imperfections as well as undefined locations of impurities with fluctuating charges, a strong suppression of current is expected with an increasing number of dots^35^,^36^.

Electron-electron interaction is naturally occurring in all electronic devices and affects transport both by scattering (such as the Auger term) and level shifts. For systems with many degrees of freedom, such as bulk or layered systems, the continuum of states justifies usually a mean-field description, so that one can apply effective single-particle levels with renormalized energies. In this case the resonances occur for different parameters, but the essential principle remains. A very successful example for this concept are quantum cascade lasers, whose operation is based on a clever design for the alignment of such single-particle levels^37^. For quantum-dot systems, however, any mean-field model is very questionable, as one replaces the interaction between quantized charges by the interaction of a charge with an averaged quantity.

This work analyzes finite bias electronic transport in both spinless and spinful triple quantum dots coupled in a serial configuration. The considered system serves as a simple model for longer arrangements such as quantum dot superlattices^38^ or possible dot-based quantum cascade lasers (QCLs)^26^,^27^. The main interest of the present work is to address the influence of different parts of the electron-electron (*ee*) interaction inside the triple dot on the electrical current. Triple quantum dots in other kind of arrangements like triangular shape was extensively studied both theoretically and experimentally^35^,^36^,^39^,^40^,^41^,^42^,^43^. We show that the Coulomb interaction between electrons opens up a large variety of different channels, which go far beyond the simple pictures of a few resonances, especially when the spin degeneracy of the levels is included. We identify two main causes: (i) Coulomb scattering provides a generic possibility for energy relaxation within the dot system. (ii) The multitude of many- particle states provides an enormous amount of different possible current paths. Additionally, this multitude of possible current paths give the possibility to study the applicability of simple Pauli master equation approach in realistic physical setup. In this work, we contrast the transport results obtained by Pauli master equation and first-order von Neumann approach^34^,^44^.

## Methods

**Figure 1:**
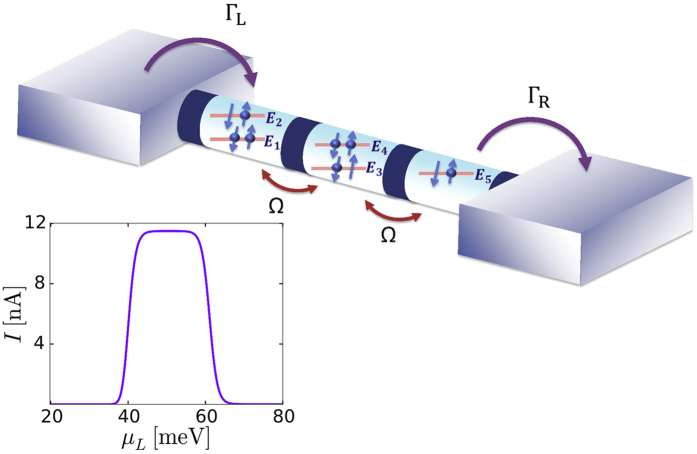
Sketch of the serial triple dot structure attached to the source and drain leads. The electrons from the source/drain lead can tunnel into the left/right dot with the rate Γ_*L*_/Γ_*R*_. The levels within the dots are coupled by the tunneling matrix Ω. Inset: Current as a function of the left Fermi level taking into account only the Coulomb scattering term *U*_*sc*_. Parameters as in Table 1.

The system under consideration consists of three serial quantum dots sandwiched between metallic leads (see Fig. 1). In this section we specify the Hamiltonian, the different approaches for transport, and the parameters used in our calculations.

### Hamiltonian

The serial triple dot is modelled by the following Hamiltonian:


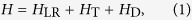


where


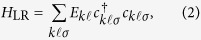


describes the source and drain leads as reservoirs with a continuum of noninteracting electrons, where 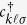 denote the electron creation operators in the leads. Here 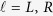 stands for the left or right lead, *σ* = ↑, ↓ denotes the spin of the electron, and 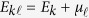 is the single particle energy of the electron in state *k*. The dispersion *E*_*k*_ in the lead is shifted by the chemical potential 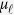 of the respective lead 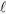. Also it is assumed that the lead states constitute a continuum with *E*_*k*_ ∈ [−*D*, *D*] having a bandwidth 2*D* and a constant density of states *ν*_*F*_ at the Fermi level. The dots are coupled to the leads by the tunneling Hamiltonian:


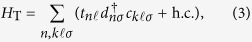


with 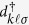 being electron creation operator in the dots, where *n* ∈ {1, 2, 3, 4, 5} labels single-particle dot states as depicted in Fig. 1. Only the levels of the left (*n* = 1, 2) and right (*n* = 5) dots are directly coupled to the left and right lead, respectively. Additionally, we assume that all couplings to the leads have the same magnitude and phase. This means that we have the following non-vanishing tunneling amplitudes:


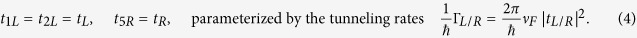


We note that for symmetric structures a sixth level in the third dot below *E*_5_ is expected. However, it is not considered here, as it hardly contributes to the current flow.

The Hamiltonian of the dots is given by


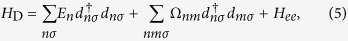


where Ω_*nm*_ describes the coupling between states *n* and *m* in neighboring dots. The electron-electron (*ee*) interaction is described by the Coulomb Hamiltonian


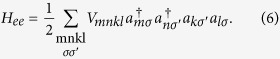


For a system that has more than one confined electron, this part plays an important role and we will discuss the different matrix elements *V*_*mnkl*_ in the following subsection. In general the Coulomb matrix elements read


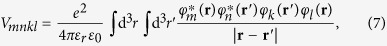


where 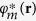 is the spatial part of the single particle state *m*, *ε*_*r*_ and *ε*_0_ are the relative and vacuum permittivity, respectively. We neglect all terms, where either *m* and *l* or *k* and *n* belong to different quantum dots, as their overlap would be vanishingly small. Furthermore, terms connecting levels of next-nearest neighboring dots are small and neglected as well. The remaining terms can be categorized into *Intradot* and *Interdot* interactions and are separately treated below.

### Intradot Interaction

For intradot interaction all the levels *mnkl* are considered to be in the same dot. By employing the normalization condition for the wave function, the direct elements can be estimated as:


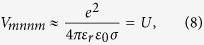


where 
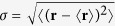
 is the standard deviation for the spatial extension of the dot wave functions. Another set of interaction matrix elements that has to be taken into account are *V*_*mnmn*_ (for *n* ≠ *m*), which act as exchange terms for equal spins and scattering terms for different spins. Trying different test wave functions, we observe


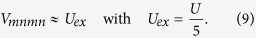


### Interdot Interaction

The direct interaction between two states in the neighboring dot can be approximated in a similar way as Eq. (8):


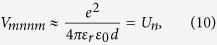


where *d* is the distance between the centers of the dots. The terms with different combinations of indices, are estimated by a Taylor expansion of 1/|**r** − **r**′| around the centers of the respective dots **R**_*i*_, **R**_*j*_, see ref. 45. Using |**R**_*i*_ − **R**_*j*_| = *d* for neighboring dots, we find


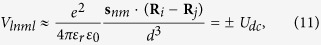



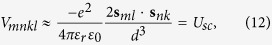


with the intradot dipole matrix element


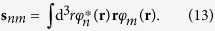


The *U*_*dc*_ and *U*_*sc*_ terms can be interpreted as a dipole-charge interaction and dipole-dipole scattering terms, respectively. The *U*_*sc*_ term is responsible for the Auger process and is crucial for the current flow in our system. The sign of *U*_*dc*_ in Eq. (11), depends on whether the charge is on the right or left side of the dipole.

### Parameter values

In order to obtain realistic and consistent parameters, we consider a particular nanostructure which is made from a nanowire containing three InAs wells (thickness 40 nm) embedded between InP barriers (thickness 3 nm). Similar structures have been recently fabricated^22^,^46^,^47^. The values of the energies *E*_*i*_ and couplings Ω_*nm*_ are estimated by a tight-binding superlattice model (see ref. 48), as outlined in the Supplementary Information. This model also provides the dipole matrix element |**s**_21_| = 8 nm, which points in the direction of the nanowire, and is used to estimate the Coulomb matrix elements as outlined above. Furthermore we use *σ *= 11 nm and *d* = 43 nm as well as *ε*_*r*_,_InAs_ = 15.^49^ In addition, the Coulomb matrix elements were calculated numerically from Eq. (7) using wave functions in cuboids (40 nm × 35 nm × 33 nm) representing each dot. This result was in good agreement (about 10% deviations) with the approximations addressed above. As we are only interested in general features, rather than in modelling a specific device, we used rounded values for all quantities in order to allow for an easy recognition of scales in the plots. The specific values are given in Table 1.

### Transport Calculation

We are interested in obtaining the current from the left to the right lead for considerably large bias. In order to do that, we diagonalize the Hamiltonian *H*_D_ (5) and get the many-particle eigenstates 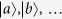 of the triple dot. Expressed in this many-particle basis the tunneling Hamiltonian *H*_T_ (3) becomes


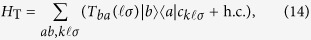



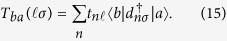


Here we used the *letter convention*: if more than one state enters an equation, then the position of the letter in the alphabet follows the particle number (for example *N*_*b*_ = *N*_*a*_ + 1, *N*_*c*_ = *N*_*a*_ + 2, *N*_*a*′_ = *N*_*a*_). In such a way the sum ∑_*bc*_ restricts to those combinations, where *N*_*c*_ = *N*_*b*_ + 1. To obtain the current through the device we use the first-order von Neumann (1 vN) approach^34^, where coherent effects are included, and a simpler Pauli master equation where only populations are considered^50^,^51^,^52^. The derivation of the governing equations is presented in Supplementary Information. The 1 vN approach treats the reduced density matrix in lowest order with respect to *H*_T_. It is conceptually similar to the Wangsness-Bloch-Redfield^44^ (also presented in Supplementary Information), albeit, the Markov limit is done in a different way. For our calculations we did not observe any differences in the current between these approaches as long as principal part integrals are neglected. We choose the temperature of the leads to be larger than the lead tunneling rate, *k*_*B*_*T*  ≫ Γ, which justifies the neglect of higher-order tunneling processes in the 1 vN and the Wangsness-Bloch-Redfield approach. Additionally, this suppresses any kind of Kondo correlations (dominating below the Kondo temperature *T*_*K*_), as *k*_*B*_*T*_*K*_ < Γ.^53^

Note that these approaches take into account electron-electron scattering by using the eigenstates of the dot-Hamiltonian. The Auger term of the Coulomb interaction with matrix element *U*_*sc*_ couples different configurations of many-particle states, which establishes connections between the leads. This does not require any relaxation terms inside the quantum-dot structure. Further relaxation processes, e. g., due to phonon-scattering, are entirely neglected here. If the emission of optical phonons is energetically not allowed, phonon scattering rates between quantum dot states are of the order of 1/ns or even smaller^54^,^55^,^56^. Such a scattering process can provide background currents of at most ≈0.1 nA. As this is smaller than the current peaks addressed in the subsequent section, it is justified to neglect these processes in our study.

## Results

In the following we show calculations for varying levels *E*_3_ and *E*_4_. This is motivated as follows: even in a nominally identical triple-dot system, growth imperfections, charged impurities and the electrostatic environment will modify the energy levels in a way which is difficult to predict. We fix the excitation energy in the left dot, *E*_2_ − *E*_1_, as a reference point and the level *E*_5_ of the right dot can be shifted by a source drain bias. However, the remaining levels *E*_3_ and *E*_4_ are less controllable, albeit there is a possibility of gating. Thus it is a central question of practical interest, for which range of parameters *E*_3_, *E*_4_ current flow occurs.

For an applied bias, standard considerations of coherent transport, such as the transmission formalism, predict current flow, if there is a state connecting both leads. If we neglect the Coulomb interaction at all, there is only a very small current unless three levels align in a row (e.g. for *E*_1_ ≈ *E*_4_ ≈ *E*_5_, a case we do not consider). For the situation in Fig. 1 one would thus not expect any elastic transport through the structure. Neglecting phonon scattering, the current would thus be zero, even if the energies *E*_3_ and *E*_4_ are varied. However, in ref. 57. it was shown, that for this situation, electron-electron scattering enables a current flow: if the levels 1 and 4 are occupied, an energy-conserving scattering event creates the simultaneous transitions 1 → 2 and 4 → 3 (for *E*_3_ − *E*_4_ = *E*_2_ − *E*_1_), with subsequent tunneling to the right lead via the state 5. The inset of Fig. 1 shows the current as a function of left Fermi level. As it is shown the current is decreasing drastically when the left Fermi level comes close to the excited state of the first dot. Then the level *E*_2_ becomes occupied, and consequently the Auger scattering process is prevented due to the Pauli blocking. This is a distinct feature of the Auger driven transport in multiple dots, which discriminates it from other possible scattering processes. Thus, scattering-assisted processes dominate.

**Figure 2:**
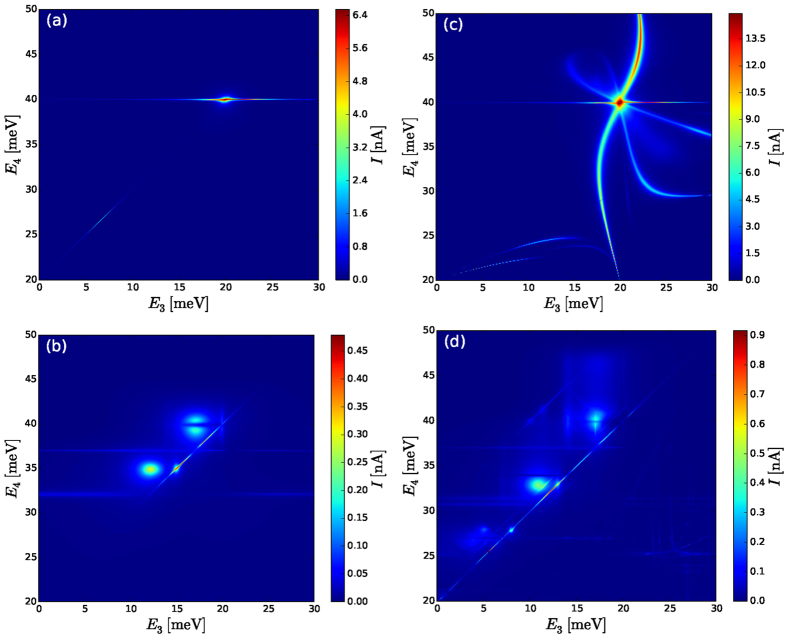
Current through the triple dot system calculated using the Pauli master equation approach. (**a**) Spin-polarized system with just scattering *U*_sc_ included [Eq. (12)]. (**b**) Spin-polarized system with all Coulomb matrix elements included. (**c**) Spinful system with just scattering *U*_sc_. (**d**) Spinful system with all Coulomb matrix elements. Parameters as in Table 1.

**Figure 3:**
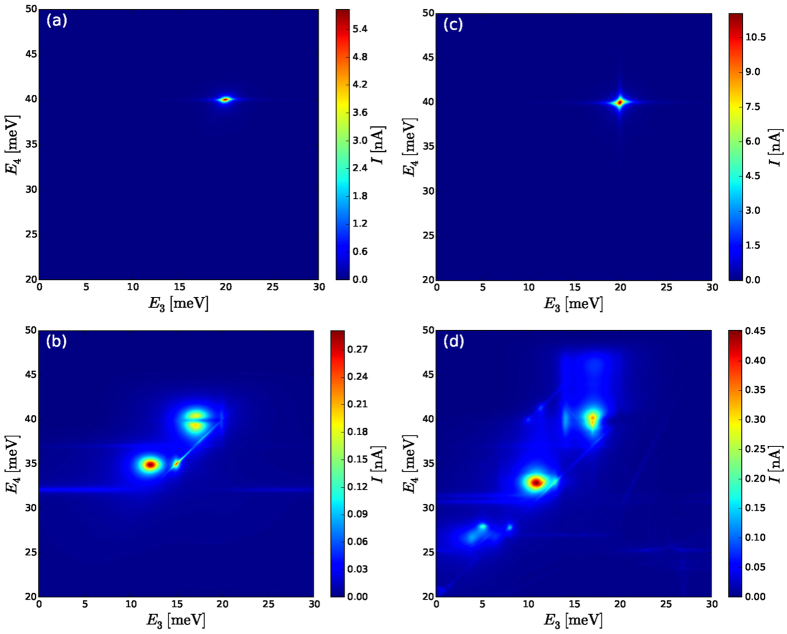
Same as Fig. 2 but with the current calculated using the 1 vN approach. (**a**) The current has a single distinct peak, where *E*_3_ ≈ *E*_5_ and *E*_4_ ≈ *E*_1_ and the resonance extends along *E*_4_ = 40 meV line. (**b**) Inclusion of all possible Coulomb elements opens up additional channels for transport leading to more current peaks. (**c**) The maximum current becomes larger by a factor of two compared to the spin-polarized case, however, now the resonance extends faintly along the *E*_4_ = 40 meV *and E*_3_ = 20 meV lines. (**d**) For spinful system additional Coulomb elements open very many channels, which leads to *significant* background current. Parameters as in Table 1.

A finite Coulomb scattering matrix element *V*_2341_ = *U*_sc_ allows for an Auger process, where one electron relaxes from level 4 to level 3, while transferring its energy to an electron being excited from level 1 to level 2. Therefore *U*_sc_ = −0.2 meV is used in all calculations, while the impact of the other Coulomb terms is neglected in some calculations in order to demonstrate their relevance. The left and right Fermi levels are fixed to be *μ*_*L*_ = 50 meV, *μ*_*R*_ = 10 meV so that the leads fill level 1 and empty the levels 2 and 5. The calculations using the Pauli master equation and the 1 vN approach are presented in Figs 2 and 3, respectively.

### Spin-polarized levels

At first we restrict to spin-polarized levels in the quantum dot. This could be achieved by having a large magnetic field in the system. Figures 2a and 3a show the current as a function of *E*_3_ and *E*_4_, when just scattering elements *U*_sc_ are included. Only for specific values of *E*_3_ and *E*_4_ the electrons are able to pass through the system. This reflects the conservation of energy both for the electron-electron scattering and for tunneling, i. e., *E*_3_ = *E*_5_ = 20 meV and *E*_4_ = *E*_1_ = 40 meV. Otherwise, the electron transport is blocked (or limited to a background current below 0.1 nA due to phonon-scattering in real structures as discussed above). Thus, any slight changes in the geometry of the dots and the configuration of the energy levels would prevent the current flow. Such a selective situation would be optimal for devices, which rely on well defined transitions, such as quantum cascade lasers.

**Figure 4:**
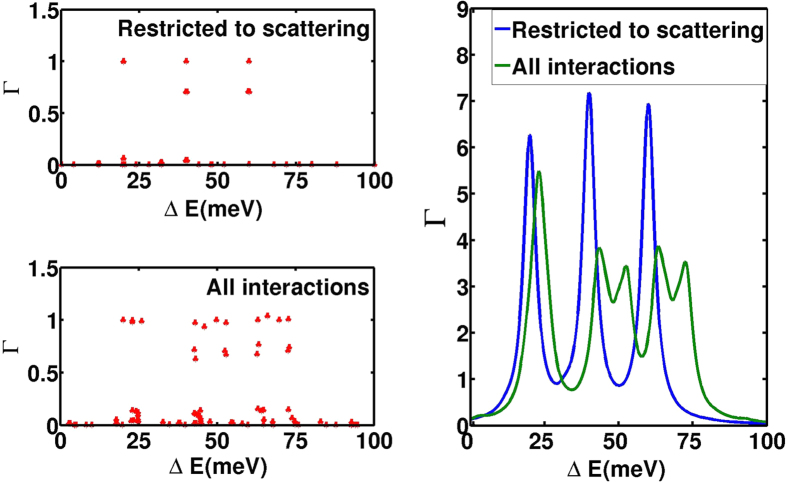
Addition energies (horizontal) for the two-particle states in the spin-polarized system. The vertical axis shows the respective coupling strength for electronic transitions from either lead. In the left panels a point is drawn for each transition. In order to resolve multiple transitions, the right panel sums Lorentzians with full width at half maximum of 5 meV with a peak given by the points in the left panel.

When *ee*-interaction with all the matrix elements is considered, we see that the current can flow for a wider range of parameters, as shown in Figs 2b and 3b. This scenario is reflected by the addition energies, where a particle can tunnel into or out of the triple dot. In Fig. 4 we display the differences in energy between all possible two and three-particle states (Δ*E* axis). In order to specify their relevance for single-particle transitions, we plot the respective transition probabilities for electrons to enter from either lead, while changing the state of the system between these states. For the case restricting to Coulomb scattering, there are only three distinct energies, where lead electrons can enter, which correspond to the energies of the levels 1, 2, and 5. In contrast, a larger variety of excitation energies is relevant if the full Coulomb interaction is taken into account. This is fully consistent with the differences between the current plots of Fig. 3a,b.

### Spinful levels

In the absence of an external magnetic field each single-particle energy level is doubly degenerate due to the Kramer’s theorem. Let us first consider the case with just scattering *U*_sc_ elements present (Fig. 3c). In a simple picture, one could expect, that the current doubles compared to the case where the levels are spin-polarized (Fig. 3a). The maximum of the current in Fig. 3c shows indeed an increase by a factor of two. Furthermore, Fig. 3c shows that the resonance faintly extends along the intersection of *E*_3_ = 20 and *E*_4_ = 40 lines. However, in a spinless case this resonance is extending just along the *E*_4_ = 40 line.

**Figure 5:**
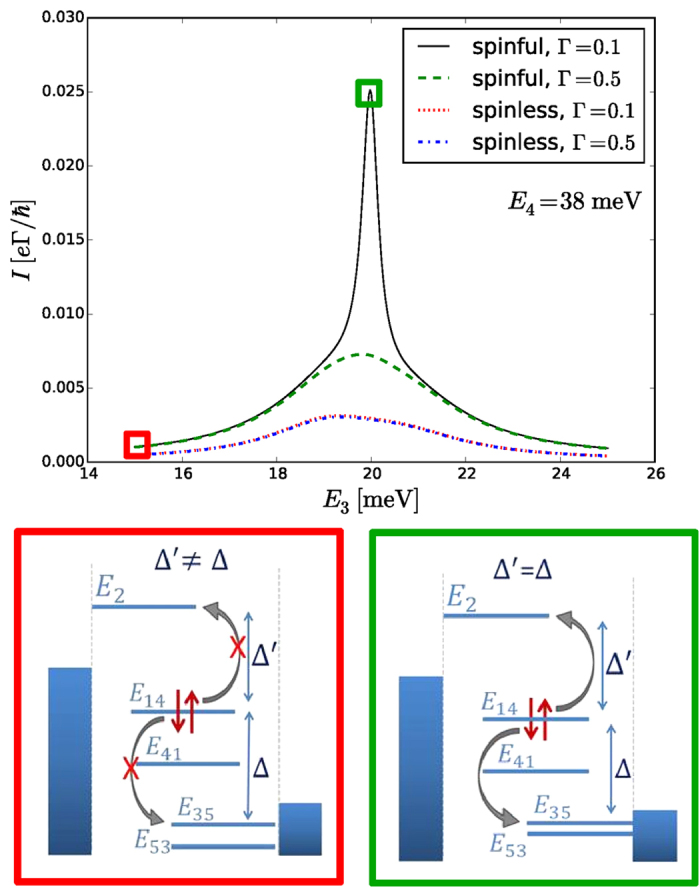
Cuts of contour plots in Fig. 3c [solid (black)] and Fig. 3a [dotted (red)] showing increased current for spinful system due to Auger process. Dashed (green) and dashed dotted (blue) gives current when the coupling Γ is increased. The left and right square panels show energy spectrum for the one-particle states at two values of energy *E*_3_. The units of Γ in the legend are meV.

In order to understand the reason of the different behavior in Fig. 3, we study a line for fixed *E*_4_ = 38 meV in Fig. 5 and corresponding eigenstates as indicated by the squares. For parameter values at the green (right) square significant current is observed for the spinful levels, but current is blocked for the spin-polarized levels. At the red (left) square current is blocked for both cases.

The five one-particle eigenstates of the Hamiltonian responsible for transport are illustrated in the green diagram in the lower diagrams of Fig. 5. There is a strong coupling between *E*_1_ and *E*_4_ as well as between *E*_3_ and *E*_5_ thus the superposition of each of these two states creates two new states with two new energies, which are referred to as *E*_1,4_, *E*_4,1_, *E*_3,5_, and *E*_5,3_. For the parameters at the right (green) square the conservation of energy is satisfied, (*E*_2_ − *E*_1,4_) ≈ (*E*_1,4_ − *E*_3,5_), which allows for Coulomb scattering. However, the state *E*_1,4_ appears as the initial state in both parts for an Auger process. Thus, the Pauli principle can only be satisfied if the level is spin degenerate. Due to this reason the electrons are able to transfer through the triple dot in a spinful system, but the current is blocked in a spin-polarized system. Spin is definitely more than a factor of two here. We note that this transport channel is rather sensitive to actual couplings to the leads. The current is *reduced* if the coupling to the leads is *increased* as can be seen from dashed (green) curve. The coherences are responsible for this surprising reduction as discussed in the next section.

Including all *ee*-interaction terms for spinful system provides a wide variety of single particle excitations and consequently resonance conditions are easier to satisfy than for the spin-polarized case addressed above. Figures 2d and 3d show the evaluated current and we observe a multitude of peaks as well as *significant* background current of ~0.15 nA for a large number of energy level combinations. As each peak relates to a different current path through the structure, it is very difficult to address or identify a specific transport path in an experiment.

### Role of coherences: comparison of 1 vN and Pauli approaches

In the following, we discuss the role of coherences, which in the 1 vN approach are defined as the off-diagonal elements of the reduced density matrix in the many-body eigenbasis of *H*_D_ (see Supplementary Information). The Pauli master equation approach neglects any such coherences, which are known to be of relevance, if Δ*E*  Γ, where Δ*E* is the separation between two relevant levels and Γ is the transition rate in units of energy. Typical examples for this situation have been already discussed in refs 58,59. In order to verify our results obtained by the Pauli master equation, we compare the 1 vN approach^34^, which keeps the coherences of the system and takes into account the tunnel transitions to the leads in lowest order. If the temperature *k*_*B*_*T* of the leads surpasses the transition rate Γ, the 1 vN approach is believed to provide reliable results for the currents, as shown by comparison with higher-order approaches^34^.

By comparing Figs 2 and 3 we see that if all interactions are included [(b) and (d)] the peak structure in the 1 vN and Pauli approaches is similar. However, the thin line of resonance for *E*_4_ ≈ *E*_3_ + 20 is substantially reduced in the more advanced 1 vN simulation. Along this line the 2-particle state occupying the levels 1 and 3, and the 2-particle state occupying levels 4 and 5 are degenerate. Via the second-order couplings they get mixed and anticross with a small splitting of Δ*E* ≈ 2 *μ*eV. This energy difference is much smaller than the transition rates Γ = 0.1, which explains, why this narrow line is mostly an artificial result in the Pauli master equation. The splitting of the 2-particle states is enhanced to Δ*E* ≈ 0.1 around *E*_4_ = 35, *E*_3_ = 15 for spinless and *E*_4_ = 32.5, *E*_3_ = 13 for spinful cases, where further 2-particle states are in resonance. This corresponds to a broader peak, which is also visible in the 1 vN simulation. If the coupling to the leads is increased, coherences become even more important, which leads to further reduction of these resonances. On the other hand, we note that the 1 vN calculations match the Pauli master equation result if the coupling to the leads Γ becomes vanishingly small compared to the energy Δ*E* splittings between the many-particle states with the same number of particles, i. e. Γ/Δ*E* → 0.

Similar considerations hold for the case when just scattering *U*_*sc*_ is included [see (a) and (c) in Figs 2 and 3]: for spin-polarized levels (a) thin lines of resonances with high current appear at *E*_4_ ≈ 40 and *E*_4_ ≈ *E*_3_ + 20 (faintly) for the Pauli master equation, which gets suppressed by coherences. However, in the spinful case (c) the Pauli master equation provides a very different scenario compared to the 1 vN approach. In Fig. 2c many resonances are pronounced, which get completely diminished by the 1 vN approach in Fig. 3c, leaving just an extended resonance along the intersection of *E*_3_ = 20 and *E*_4_ = 40 lines.

### Neglect of principal part terms

**Figure 6:**
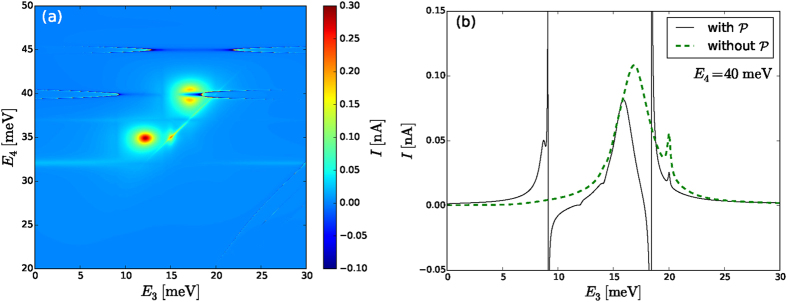
Current for spin-polarized system with all interactions calculated using the 1 vN approach [similar to Fig. 3b], when the principal part 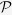 integrals are included (see Eq. (S20) in Supplementary Information). (**a**) Due to divergent current the values below −0.1 nA and above +0.3 nA are filtered. (**b**) Cut showing the comparison of calculated current with and without the principal parts.

In the 1 vN approach calculations of Figs 1, 2, 3, 4, 5 we have neglected the principal part integrals (see Eq. (S20) in Supplementary Information). At the resonance position 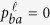 this integral gives a logarithmic divergence, which is cut-off by the temperature. So it could be expected that for large enough temperature these terms should not be problematic. However, in Fig. 6, we show a calculation for spin-polarized quantum dots, with all interactions and the principal part integrals included. We see that regions of negative current appear (flow against the bias), and at particular points the current also has divergencies (see Fig. 6b). At these points of divergent current the positivity of the populations Φ_*bb*_ is highly violated and the elements Φ_*bb*′_ acquire divergent structure as well. This unphysical behavior of the current motivates the neglect of the principal part integrals.

Finally, the principal parts have the structure of the renormalization of the energy spectrum (Lamb shift^60^) as can be seen from perturbation theory for energies^61^ or real parts of self-energies in Green’s function formalism^62^. This suggest that this type of terms should be resummed to get an effective Hamiltonian in which new eigenspectrum of the quantum dots is obtained and the energy differences *E*_*b*_ − *E*_*a*_ are renormalized^63^,^64^.

## Discussion

The main results are collected in Fig. 3, which displays the current through both spin-polarized and spinful triple quantum dot system, calculated using the 1 vN approach. For the single spin case restricting just to scattering *U*_sc_, a single peak is seen in Fig. 3a, which is easily predicted by standard single particle states. Such resonances are well known and are commonly used for device design. However, taking into account spin degeneracy of the states and all interactions, Fig. 3d displays an entirely different picture, where current flow is spread over a wide range of parameters where unexpected paths become of relevance. As a consequence, designing devices based on multiple quantum dots is questionable, if one restricts to single-particle models even if the mean-field is included. For example, for dot-based quantum cascade lasers, it is difficult to accomplish the desired specificity of electron injection into the upper laser level. On the other hand, the multitude of channels creates *significant* background current for wide range of energy level configurations, which makes transport in multiple quantum-dot devices less sensitive to size fluctuations and random charges.

We have compared the Pauli master equation approach, which neglects coherences, and the 1 vN approach where all density matrix elements of the reduced quantum dot system are taken into consideration. It was shown that the qualitative picture of resonances is correctly captured by the simpler Pauli master equation. However, there are cases where the current is highly overestimated by this approach and this can be seen by comparing Figs 2c and 3c. These points are traced back to specific level configurations with the lead coupling larger than the energy splitting, i. e., Γ > Δ*E*, where coherences strongly reduce the current. This also allows for situations where the current drops with increasing lead coupling Γ as shown Fig. 5. Additionally, the relevance of principal part integrals in the 1 vN approach was examined and it was shown that it can lead to highly unphysical results (current flowing against the bias), which suggests that neglecting such terms is reasonable.

## Additional Information

**How to cite this article**: Goldozian, B. *et al.* Transport in serial spinful multiple-dot systems: The role of electron-electron interactions and coherences. *Sci. Rep.* **6**, 22761; doi: 10.1038/srep22761 (2016).

## Supplementary Material

Supplementary Information

## Figures and Tables

**Table 1:** Parameters used in the calculations if not mentioned otherwise.

*E*_1_ = 40	*U* = 10	Γ_*L*_ = 0.1	Ω_13_ = −0.05
*E*_2_ = 60	*U*_*ex*_ = 2	Γ_*R*_ = 0.1	Ω_14_ = 0.1
*E*_3_ = 20	*U*_*n*_ = 3	*μ*_*L*_ = 50	Ω_23_ = 0.1
*E*_4_ = 40	*U*_*SC*_ = −0.2	*μ*_*R*_ = 10	Ω_24_ = 0.2
*E*_1_ = 20	*U*_*dc*_ = −0.5	*k*_B_*T* = 1	Ω_35_ = 0.1
		*D* = 10^4^	Ω_45_ = 0.2

All energies are in meV.
