# Supplementary material for: Transport in serial spinful multiple-dot systems: The role of electron-electron interactions and coherences
Source: Sci Rep. 2016 Mar 7;6:22761. doi: 10.1038/srep22761 (PMC4780117; doi:10.1038/srep22761)
Supplement: Supplementary Information [file srep22761-s1.pdf]

Supplementary Information for "Transport in serial spinful multiple-dot systems: The role of electron-electron interactions and coherences"

Bahareh Goldozian,[1] Fikeraddis A. Damtie,[1] Gediminas Kiršanskas,[1] and Andreas Wacker[1]

[1]*Mathematical Physics and NanoLund, Lund University, Box 118, S-22100 Lund, Sweden*

(Dated: February 2, 2016)

I. FIRST-ORDER VON NEUMANN (1VN) APPROACH

The dynamics of the full density matrix ρ of the system is described by the von Neumann equation:

$$i\hbar\frac{\partial}{\partial t}\rho = [H,\rho]. \tag{S1}$$

We solve the above equation approximately using the 1vN approach.[1–3] In this approach only the density matrix elements, which connect the states differing by just one electron or hole excitation, are considered. Additionally, such a treatment is valid if the temperature T is larger than the tunneling rates, $T \gg \Gamma$. In the other limit, $T \ll \Gamma$, different approaches based on numerical renormalization group,[4] quantum Monte Carlo simulations,[5] or higher order expansions should be used.[1,6–8] The density matrix elements are defined as

$$\rho^{[n]}_{ag,bg'} = \langle ag|\rho|bg'\rangle, \tag{S2}$$

where $|bg\rangle = |b\rangle \otimes |g\rangle$, with $|b\rangle$ denoting the eigenstate of the dot Hamiltonian (5) and $|g\rangle$ denoting the eigenstate of the lead Hamiltonian (2). Here the label n provides the number of electron or hole excitations needed to transform $|g\rangle$ into $|g'\rangle$. For example, we will consider matrix elements of the type

$$\begin{aligned}\rho^{[0]}_{bg,b'g} &= \langle bg|\rho|b'g\rangle,\\ \rho^{[1]}_{bg-\kappa,ag} &= \langle bg-\kappa|\rho|ag\rangle.\end{aligned} \tag{S3}$$

Here we have introduced the following notation

$$\kappa \equiv k,\ \ell,\ \sigma; \tag{S4}$$

$$\begin{aligned}|bg+\kappa\rangle &= |b\rangle \otimes c^\dagger_\kappa|g\rangle,\\ |bg-\kappa\rangle &= |b\rangle \otimes c_\kappa|g\rangle.\end{aligned} \tag{S5}$$

By neglecting all the density matrix elements with more than one electron or hole excitation $n \geq 2$ from Eq. (S1) we obtain the equations

$$\begin{aligned}i\hbar\frac{\partial}{\partial t}\rho^{[0]}_{bg,b'g} &= (E_b - E_{b'})\rho^{[0]}_{bg,b'g}\\ &+ T_{ba_1}(\kappa_1)\rho^{[1]}_{a_1g+\kappa_1,b'g}(-1)^{N_{a_1}}\\ &+ T_{bc_1}(\kappa_1)\rho^{[1]}_{c_1g-\kappa_1,b'g}(-1)^{N_b}\\ &- \rho^{[1]}_{bg,c_1g-\kappa_1}(-1)^{N_{b'}}T_{c_1b'}(\kappa_1)\\ &- \rho^{[1]}_{bg,a_1g+\kappa_1}(-1)^{N_{a_1}}T_{a_1b'}(\kappa_1),\end{aligned} \tag{S6}$$

$$\begin{aligned}i\hbar\frac{\partial}{\partial t}\rho^{[1]}_{cg-\kappa,bg} &\approx (E_c - E_\kappa - E_b)\rho^{[1]}_{cg-\kappa,bg}\\ &+ T_{cb_1}(\kappa)\rho^{[0]}_{b_1g,bg}(-1)^{N_{b_1}}\langle g|c^\dagger_\kappa c_\kappa|g\rangle\\ &- \rho^{[0]}_{cg-\kappa,c_1g-\kappa}(-1)^{N_b}T_{c_1b}(\kappa).\end{aligned} \tag{S7}$$

Here N_b denotes the number of electrons in the state $|b\rangle$. Note that all indices with subscript 1 like $a_1,\ c_1,\ \kappa_1$ are summed over and the *letter convention* introduced in the main text is used. Also the matrix element $\langle g|c^\dagger_\kappa c_\kappa|g\rangle$ in the second term of the right-hand side of Eq. (S7) corresponds to the requirement that there is an electron in the single particle state corresponding to κ. Additionally, phase factors like $(-1)^{N_b}$ appear due to order exchange of the lead operators with the dot operators, i.e., $c_\kappa(|b\rangle \otimes |g\rangle) = (-1)^{N_b}|b\rangle \otimes c_\kappa|g\rangle$.

Summing Eqs. (S6) and (S7) over all the lead states $|g\rangle$ we get

$$\begin{aligned}i\hbar\frac{\partial}{\partial t}\Phi^{[0]}_{bb'} &= (E_b - E_{b'})\Phi^{[0]}_{bb'}\\ &+ T_{ba_1}(\kappa_1)\Phi^{[1]}_{a_1b'}(\kappa_1) + T_{bc_1}(\kappa_1)\Phi^{[1]}_{c_1b'}(\kappa_1)\\ &- \Phi^{[1]}_{bc_1}(\kappa_1)T_{c_1b'}(\kappa_1) - \Phi^{[1]}_{ba_1}(\kappa_1)T_{a_1b'}(\kappa_1)\end{aligned} \tag{S8}$$

$$\begin{aligned}i\hbar\frac{\partial}{\partial t}\Phi^{[1]}_{cb}(\kappa) &\approx (E_c - E_\kappa - E_b)\Phi^{[1]}_{cb}(\kappa)\\ &+ T_{cb_1}(\kappa)\Phi^{[0]}_{b_1b}f_\kappa - \Phi^{[0]}_{cc_1}T_{c_1b}(\kappa)f_{-\kappa},\end{aligned} \tag{S9}$$

where we introduced the following notation

$$\begin{aligned}\Phi^{[0]}_{bb'} &= \sum_g \rho^{[0]}_{bg,b'g},\\ \Phi^{[1]}_{cb}(\kappa) &= \sum_g \rho^{[1]}_{cg-\kappa,bg}(-1)^{N_b},\\ \Phi^{[1]}_{bc}(\kappa) &= \left[\Phi^{[1]}_{cb}(\kappa)\right]^*,\\ f_\kappa &\equiv f_{k\ell} = (\exp[E_k/k_\mathrm{B}T_\ell]+1)^{-1},\\ f_{-\kappa} &\equiv 1 - f_{k\ell}.\end{aligned} \tag{S10}$$

Here we have also assumed that the electrons in the leads are *thermally distributed* according to the Fermi-Dirac distribution f and that this distribution is not affected by the coupling to the quantum dots. This assumption leads to the following relations:

$$\begin{aligned}\sum_g \rho^{[0]}_{bg,b'g}\langle g|c^\dagger_\kappa c_\kappa|g\rangle &\approx f_\kappa\Phi^{[0]}_{bb'},\\ \sum_g \rho^{[0]}_{bg-\kappa,b'g-\kappa} &\approx f_{-\kappa}\Phi^{[0]}_{bb'}.\end{aligned} \tag{S11}$$

For the stationary state we assume the conditions

$$i\hbar\frac{\partial}{\partial t}\Phi^{[0]}_{bb'}=0,\quad i\hbar\frac{\partial}{\partial t}\Phi^{[1]}_{cb}(\kappa)=0, \tag{S12}$$

which allow to write $\Phi^{[1]}$ in terms of $\Phi^{[0]}$ as

$$\Phi^{[1]}_{cb}(\kappa)=\frac{T_{cb_1}(\kappa)\Phi^{[0]}_{b_1b}f_\kappa-\Phi^{[0]}_{cc_1}T_{c_1b}(\kappa)f_{-\kappa}}{E_\kappa-E_c+E_b+i\eta}. \tag{S13}$$

Here we have added a positive infinitesimal $\eta=+0$ to ensure a proper decay of initial conditions, which can be seen by formally integrating Eq. (S9):

$$\begin{aligned}\Phi^{[1]}_{cb}(\kappa,t)=&\frac{1}{i\hbar}\int_{-\infty}^{t}\mathrm{d}t'e^{i(E_\kappa-E_c+E_b+i\eta)(t-t')/\hbar}\\&\times\left(T_{cb_1}(\kappa)\Phi^{[0]}_{b_1b}(t')f_\kappa-\Phi^{[0]}_{cc_1}(t')T_{c_1b}(\kappa)f_{-\kappa}\right).\end{aligned} \tag{S14}$$

After performing a *Markov* approximation in the above integral, $\Phi^{[0]}_{bb'}(t')\approx\Phi^{[0]}_{bb'}(t)$, and setting $t\to+\infty$ we also obtain Eq. (S13). We note that there is another possibility to choose the time dependence of $\Phi^{[0]}$:

$$\Phi^{[0]}_{bb'}(t')\approx e^{i(E_b-E_{b'})(t-t')/\hbar}\Phi^{[0]}_{bb'}(t). \tag{S15}$$

In this case we obtain,

$$\begin{aligned}\Phi^{[1]}_{cb}(\kappa)=&\frac{T_{cb_1}(\kappa)\Phi^{[0]}_{b_1b}f_\kappa}{E_\kappa-E_c+E_{b_1}+i\eta}\\&-\frac{\Phi^{[0]}_{cc_1}T_{c_1b}(\kappa)f_{-\kappa}}{E_\kappa-E_{c_1}+E_b+i\eta}.\end{aligned} \tag{S16}$$

which together with Eq. (S8) provides the *Wangsness-Bloch-Redfield* approach.[9] The denominators in Eq. (S16) differ from Eq. (S13) if non-diagonal density matrix elements $\Phi^{[0]}_{bb'}$ are relevant. The oscillatory behavior in Eq. (S15) is suggested by the first right-hand side term in Eq. (S8).

After combining Eqs. (S8), (S12), and (S13) we get the 1vN approach equations for the steady state:

$$\begin{aligned}0=&\Phi_{bb'}(E_b-E_{b'})\\&+\sum_{b''\ell}\Phi_{bb''}\Big[\sum_a\Gamma^\ell_{b''a,ab'}I^{\ell-}_{ba}-\sum_c\Gamma^\ell_{b''c,cb'}I^{\ell+*}_{cb}\Big]\\&+\sum_{b''\ell}\Phi_{b''b'}\Big[\sum_c\Gamma^\ell_{bc,cb''}I^{\ell+}_{cb'}-\sum_a\Gamma^\ell_{ba,ab''}I^{\ell-*}_{b'a}\Big]\\&+\sum_{aa'\ell}\Phi_{aa'}\Gamma^\ell_{ba,a'b'}[I^{\ell+*}_{b'a}-I^{\ell+}_{ba'}]\\&+\sum_{cc'\ell}\Phi_{cc'}\Gamma^\ell_{bc,c'b'}[I^{\ell-*}_{c'b}-I^{\ell-}_{cb'}].\end{aligned} \tag{S17}$$

Additionally, we impose the normalisation condition for diagonal density matrix elements:

$$\sum_b\Phi_{bb}=1. \tag{S18}$$

Here in Eq. (S17) the tunneling rate matrix Γ is defined as

$$\Gamma^\ell_{ba,a'b'}=2\pi\nu_F\sum_\sigma T_{ba}(\ell\sigma)T_{a'b'}(\ell\sigma), \tag{S19}$$

and the following integral was introduced

$$\begin{aligned}2\pi I^{\ell\pm}_{ba}&=\mathcal{P}\int_{-D}^{D}\frac{\mathrm{d}Ef(\pm E)}{E-p^\ell_{ba}}-i\pi f(\pm p^\ell_{ba})\theta(D-|p^\ell_{ba}|),\\p^\ell_{ba}&=E_b-E_a-\mu_\ell,\\f(E)&=(\exp[E/k_\mathrm{B}T]+1)^{-1},\end{aligned} \tag{S20}$$

which appears after performing the k-sums using a flat density of states approximation, i. e., $\sum_k\to\nu_F\int_{-D}^{D}\mathrm{d}E$, with ν_F denoting the density of states at the Fermi level and $2D$ denoting the bandwidth of the leads. In our calculations we assume that the bandwidth of the leads is the largest energy scale. Note that, in the limit $D\to+\infty$ the results become bandwidth independent[10] and that is what we also have checked in our numerical simulations. We note that the 1vN approach does not include any broadening effects of the quantum dot levels due to leads, which in principle could be included by different means (e.g., see Refs. [1, 7, 11, and 12]).

Finally, we are interested in the current going from the lead ℓ into the quantum dots, which is given by

$$\begin{aligned}I_\ell(t)&=e\sum_{k\sigma}\frac{\partial}{\partial t}\langle c^\dagger_{k\ell\sigma}(t)c_{k\ell\sigma}(t)\rangle\\&=\frac{2e}{\hbar}\sum_{k\sigma}\mathrm{Im}[T_{bc}(\ell\sigma)\Phi^{[1]}_{cb}(k\ell\sigma)].\end{aligned} \tag{S21}$$

Here $A(t)=e^{iHt}Ae^{-iHt}$ denotes the Heisenberg evolution of an operator A. In the steady state the current is obtained from Eq. (S21) in terms of $\Phi^{[0]}_{b'b}$ as

$$I_\ell=\frac{2e}{\hbar}\sum_{cb}\mathrm{Im}\Big[\sum_{b'}\Gamma^\ell_{bc,cb'}I^{\ell+}_{cb}\Phi^{[0]}_{b'b}-\sum_{c'}\Gamma^\ell_{bc,c'b}I^{\ell-}_{cb}\Phi^{[0]}_{cc'}\Big], \tag{S22}$$

which is the main output presented in the paper. For all the calculations (except for Fig. 6) the principal part integral $\mathcal{P}$ in Eq. (S20) is neglected. The motivation for neglecting these terms is given in Section **"Neglect of principal part terms"** of the main text.

II. PAULI MASTER EQUATION

The Pauli master equation can be obtained from the 1vN approach by neglecting the coherences $\Phi_{bb'}$, $b\neq b'$. In such a case for the populations $P_b=\Phi_{bb}$ we obtain the equations:

$$\begin{aligned}&\sum_{a\ell}\left[P_a\Gamma^\ell_{a\to b}f(+p^\ell_{ba})-P_b\Gamma^\ell_{b\to a}f(-p^\ell_{ba})\right]\\&+\sum_{c\ell}\left[P_c\Gamma^\ell_{c\to b}f(-p^\ell_{cb})-P_b\Gamma^\ell_{b\to c}f(+p^\ell_{cb})\right]=0,\end{aligned} \tag{S23}$$

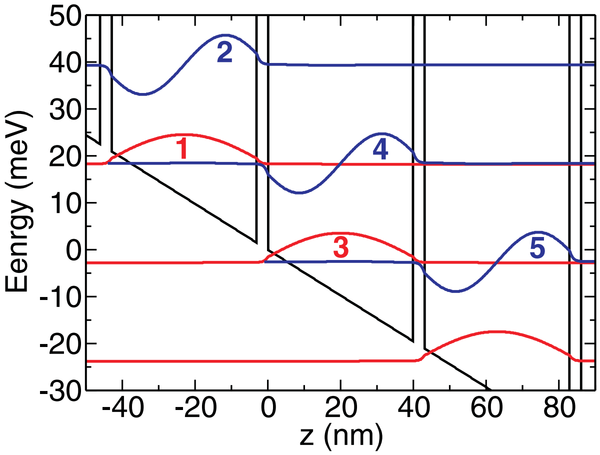

Fig. S1. Wannier levels for the superlattice structure at a voltage drop of 21 meV per period. We use just three periods of this structure to model single particle levels in the serial triple dot.

where we have denoted $\Gamma^{\ell}_{a\to b} = \Gamma^{\ell}_{ab,ba} = \Gamma^{\ell}_{b\to a} = \Gamma^{\ell}_{ba,ab}$. Using the populations P_b the steady states current is expressed as

$$I_\ell = \frac{e}{\hbar}\sum_{ab}[P_a\Gamma^{\ell}_{a\to b}f(+p^{\ell}_{ba}) - P_b\Gamma^{\ell}_{b\to a}f(-p^{\ell}_{ba})]. \quad \text{(S24)}$$

III. MOTIVATION FOR PARAMETER VALUES

We consider a superlattice structure with 40 nm InAs wells ($m_{\text{eff}} = 0.026$) and 3 nm InP barriers ($m_{\text{eff}} = 0.08$, $\Delta E_c = 0.6$ eV) providing the period $d = 43$ nm. The serial triple dot is described by just three periods of such a structure. A standard calculation (see, for example, Ref. [13]) provides the minibands $E_\nu(q) \approx E_\nu + 2T_\nu \cos(qd)$ with $E_a = 7$ meV, $T_a = -0.058$ meV, $E_b = 28$ meV, and $T_b = -0.23$ meV. Figure S1 displays the corresponding Wannier functions, where an electric field of 21meV$/d$ is applied, so that the ground state a of one well is in resonance with the excited state b of the neighboring well. The tunnel couplings between Wannier states from the same band ν in neighboring periods is T_ν. Thus we identify

- $\Omega_{13} = T_a = -0.058$ meV

- $\Omega_{24} = \Omega_{45} = T_b = 0.23$ meV

Furthermore, we obtain numerically the dipole matrix elements $s_{nm} = s_{mn} = \int \mathrm{d}z\varphi^*_n(z)z\varphi_m(z)$ between the states. The non-vanishing values are $s_{21} = s_{43} = 8.16$ nm (which is also used for the calculation of Coulomb matrix elements for the interdot interaction after making a dipole expansion, see Ref. [14]) and $s_{41} = s_{32} = s_{53} = -0.21$ nm. For the operation bias this provides the tunnel couplings between states from different bands $\Omega_{nm} = -eFs_{nm}$ with the values

- $\Omega_{41} = \Omega_{53} = \Omega_{32} = 0.103$ meV

We note, that the same procedure is used for simulating quantum cascade lasers, where we obtain quantitative agreement for a variety of samples.[15,16]

1 J. N. Pedersen and A. Wacker, Phys. Rev. B **72**, 195330 (2005).
2 J. N. Pedersen, B. Lassen, A. Wacker, and M. H. Hettler, Phys. Rev. B **75**, 235314 (2007).
3 J. N. Pedersen and A. Wacker, Physica E **42**, 595 (2010).
4 F. B. Anders, Phys. Rev. Lett. **101**, 066804 (2008).
5 J. E. Han and R. J. Heary, Phys. Rev. Lett. **99**, 236808 (2007).
6 S. Hershfield, Phys. Rev. Lett. **70**, 2134 (1993).
7 J. Jin, X. Zheng, and Y. Yan, J. Chem. Phys. **128**, 234703 (2008).
8 H. Schoeller, Eur. Phys. J. Spec. Top. **168**, 179 (2009).
9 H.-P. Breuer and F. Petruccione, *Open Quantum Systems* (Oxford University Press, Oxford, 2006).
10 F. D. M. Haldane, Phys. Rev. Lett. **40**, 416 (1978).
11 A. Dorda, M. Nuss, W. von der Linden, and E. Arrigoni, Phys. Rev. B **89**, 165105 (2014).
12 L. Chen, T. Hansen, and I. Franco, J. Phys. Chem. C **118**, 20009 (2014).
13 A. Wacker, Phys. Rep. **357**, 1 (2002).
14 B. Lassen and A. Wacker, Phys. Rev. B **76**, 075316 (2007).
15 M. Lindskog, J. M. Wolf, V. Trinite, V. Liverini, J. Faist, G. Maisons, M. Carras, R. Aidam, R. Ostendorf, and A. Wacker, Appl. Phys. Lett. **105**, 103106 (2014).
16 E. Dupont, S. Fathololoumi, Z. R. Wasilewski, G. Aers, S. R. Laframboise, M. Lindskog, S. G. Razavipour, A. Wacker, D. Ban, and H. C. Liu, J. Appl. Phys. **111**, 073111 (2012).
